# Text Mining of Adverse Events in Clinical Trials: Deep Learning Approach

**DOI:** 10.2196/28632

**Published:** 2021-12-24

**Authors:** Daphne Chopard, Matthias S Treder, Padraig Corcoran, Nagheen Ahmed, Claire Johnson, Monica Busse, Irena Spasic

**Affiliations:** 1 School of Computer Science & Informatics Cardiff University Cardiff United Kingdom; 2 Centre for Trials Research Cardiff University Cardiff United Kingdom

**Keywords:** natural language processing, deep learning, machine learning, classification

## Abstract

**Background:**

Pharmacovigilance and safety reporting, which involve processes for monitoring the use of medicines in clinical trials, play a critical role in the identification of previously unrecognized adverse events or changes in the patterns of adverse events.

**Objective:**

This study aims to demonstrate the feasibility of automating the coding of adverse events described in the narrative section of the serious adverse event report forms to enable statistical analysis of the aforementioned patterns.

**Methods:**

We used the Uniﬁed Medical Language System (UMLS) as the coding scheme, which integrates 217 source vocabularies, thus enabling coding against other relevant terminologies such as the International Classification of Diseases–10th Revision, Medical Dictionary for Regulatory Activities, and Systematized Nomenclature of Medicine). We used MetaMap, a highly configurable dictionary lookup software, to identify the mentions of the UMLS concepts. We trained a binary classifier using Bidirectional Encoder Representations from Transformers (BERT), a transformer-based language model that captures contextual relationships, to differentiate between mentions of the UMLS concepts that represented adverse events and those that did not.

**Results:**

The model achieved a high F1 score of 0.8080, despite the class imbalance. This is 10.15 percent points lower than human-like performance but also 17.45 percent points higher than that of the baseline approach.

**Conclusions:**

These results confirmed that automated coding of adverse events described in the narrative section of serious adverse event reports is feasible. Once coded, adverse events can be statistically analyzed so that any correlations with the trialed medicines can be estimated in a timely fashion.

## Introduction

### Background

Modern health care is associated with increased costs and broad-reaching variations in care and outcomes across the global population. The provision of evidence-based health care is a critical priority for users, providers, and policy makers alike. The systematic and high-quality conduct of clinical trials is critical for the development of clinical guidance to inform evidence-based practice. Pharmacovigilance and safety reporting are among the most important aspects of the conduct of clinical trials. This is relevant to all clinical trials in which the benefit or harm must be fully established before any intervention or medicinal product is adopted.

Pharmacovigilance and safety reporting provide the basis for ensuring clinical trial participant safety and good research practice. It involves processes for monitoring the use of medicines or interventions in clinical trials. It has a critical role in the identification of previously unrecognized adverse events or changes in the patterns of adverse events. It is also relevant to the assessment of the risks and benefits of medicines or interventions to determine what action, if any, is needed to improve their safe use.

An adverse event is any untoward medical occurrence in a participant to whom a medicinal product has been administered, including occurrences that are not necessarily caused by or related to the administered product. A serious adverse event (SAE) is any untoward medical occurrence that, at any dose, results in death, is life-threatening, requires inpatient hospitalization or causes prolongation of existing hospitalization, results in persistent or significant disability or incapacity, or comprises a congenital anomaly or birth defect. Early detection of unknown adverse events, reactions, interactions, and an increase in the frequency of (known) adverse events is a key element of the pharmacovigilance and safety process. Provision of up-to-date information on adverse events to health care professionals, researchers, and regulatory bodies contributes to the assessment of benefit, harm, effectiveness, and risk of the intervention, thus advancing their safe, rational, and more effective (including cost-effective) use.

In multicenter noncommercial clinical trials conducted in the United Kingdom, the SAE reporting requirements are detailed in the trial protocol, and the principal investigators at National Health Service sites are responsible for reporting SAEs to the coordinating clinical trial unit (CTU) for an assessment of the seriousness, causality, and expectedness as delegated by the clinical trial sponsor. An SAE report includes an event term and additional signs and symptoms in a narrative. The narrative is reported by a physician during their medical assessment of the event. The report is then reviewed by a central CTU reviewer to assess any potential causal relationship with the trial drug. Each narrative is reviewed as a single report. The narratives are typically received from sites as paper records. These are logged electronically in the safety databases by the CTU pharmacovigilance team for the relevant national competent authorities (eg, the UK Medicines and Health Care Products Regulatory Agency or European Medicines Agency). The reports are searchable on request and subject to appropriate regulatory permissions. There is now a clear recognition of the potential for artificial intelligence in safety case management to identify relationships and signals [1]. Although these approaches may be implemented in commercial settings and within competent authorities, such methods for classifying and categorizing data are not yet standardized or explicit across noncommercial pharmacovigilance settings.

It is possible that the narrative contains additional adverse events or toxicities that are not coded as additional events and are captured in the narrative only. However, there is no mechanism for the detection of safety signals across individual reports or individual trials and, thus, there is no possibility for early detection of worrying trends. This is particularly the case for toxicities for which reconciliation with the clinical database would be advantageous. Such a tool would facilitate the cross-checking of toxicities recorded in the narrative of the SAE form with those recorded in the trial database, which is currently only feasible if automated. Although these approaches may be used in commercial trial settings, they would not always be used in the public domain simply because of the nature of the drug licensing pathway.

This study seeks to use text mining to automatically identify and code adverse events from the narrative sections of SAE reports in clinical trials of investigational medicinal products coordinated by a noncommercial CTU, with the aim of unlocking narrative evidence for further statistical analysis. Although such an analysis is beyond the scope of this study, it would serve to monitor the patterns of adverse events at the cohort level rather than singular adverse events. Owing to their narrative nature, such an analysis cannot be conducted directly on the content of SAE reports.

### Related Work

Text mining has been used to identify adverse events from a variety of data sources, including spontaneous reporting systems, medical literature, electronic health records, and user-generated content on the internet [2]. The problem of mining adverse events in text has been approached from different angles. Most commonly, it has been defined as a text classification problem, where a piece of text, either an entire document or its part (eg, an individual sentence), is mapped to ≥1 predefined class that correspond to a type of adverse event or its property. Some approaches target a specific adverse event such as anaphylaxis and perform simple binary classification with respect to the presence of the event considered [3]. Other examples target a range of drugs and use documents that mention them to train a binary classifier with respect to their safety, using an existing watch list of drugs that have an active safety alert posted on the US Food and Drug Administration website [4].

In terms of semantics, adverse events are compatible with signs and symptoms. When a dictionary-based method is used to extract such instances, a binary classifier is needed to differentiate between the signs and symptoms that correspond to adverse events and those associated with the underlying diagnosis [5]. Along similar lines, when an adverse event is associated with medication, a system is needed to support safety evaluators in identifying reports that may demonstrate causal relationships with the suspect medications. To this end, it has been shown that a binary classifier can be trained to successfully differentiate between 2 causality categories: certain, probable, or possible versus unlikely or unassessable [6]. Multifaceted classification can be performed to identify additional properties of an adverse event, for example, temporal (historical or present), categorical (assertive, hypothetical, retrospective, or a general discussion), and contextual (deduced or explicitly stated) [7].

Alternatively, the problem of identifying adverse events can be defined as that of information extraction [8]. More specifically, we can differentiate between entity and relationship extraction. Here, the goal of entity extraction is to identify a text sequence that describes an adverse event. Therefore, it can also be viewed as a sequence labeling problem [9-11]. In addition, the text sequence can be mapped to a relevant dictionary such as the Medical Dictionary for Regulatory Activities [12,13] or the Uniﬁed Medical Language System (UMLS) [9,14]. Such normalization of named entities to standardized identifiers is especially relevant when processing text originating from social media, whose language tends to be highly colloquial [4,9,10,13-17].

When multiple medicines are considered, 2 types of named entities need to be extracted—medicines and adverse events—and additional reasoning needs to be performed to extract a relationship between the two [7,17,18]. Further statistical analysis can be applied to such pairs to measure the strength of such associations [18]. Information of interest can be extracted using pattern-matching approaches, where patterns are typically modeled using regular expressions [7,12,19]. Alternatively, frequent patterns of language for expressing opinions about medications can be learned automatically using association rule mining by considering sentences as transactions and the words in a sentence as items in the transactions [15].

Specific methods chosen to mine adverse events from text depend on the way the text mining problem is posed. Typical approaches chosen for text classification include rule-based methods [3,7,14,20] and supervised machine learning [3-6,16,21]. A range of machine learning methods has been used, including naive Bayes, support vector machines, random forests, maximum entropy, and logistic regression. On occasion, ensemble learning has been used to improve classification performance by integrating multiple models using methods such as bagging, majority voting, weighted averaging, and stacked generalization [4,17,21]. The different types of lexical, syntactic, and semantic features have been used by the classification algorithms. Lexical features include n-grams [4,16], context windows [17], and lexicon matches [16]. Typically, syntactic features include part-of-speech tags, negation, syntactic dependencies, and syntactic functions [16,17,21]. Semantic features are either based on external sources such as the UMLS, PubChem, or DrugBank [16,17,20,22] or manually engineered [4-7]. Other used features were based on sentiment polarities [4,16] and topic modeling [16]. A few examples of using feature selection methods include binormal separation [4] and information gain [17].

Finally, approaches chosen to address adverse event mining as a sequence labeling problem include conditional random fields (CRFs) [9,23] and, more recently, neural networks (NNs) [21,22], including recurrent NNs [10] and long short-term memory (LSTM) [24], which outperformed CRFs. For best results, bidirectional LSTM is combined with CRF [11,25-29]. Most approaches used word embeddings, which represent words as meaningful real-valued vectors of configurable dimensions learned automatically from a large corpus based on their co-occurrence using methods such as word2vec [22,27], fastText [24], and GloVe [30]). Traditional bag-of-words (BOW) approaches tend to struggle with unseen or rare words. Word embeddings that are pretrained on a large corpus remedy this problem and, consequently, boost recall (R).

The aforementioned word-embedding models generate a single embedding for each word, thus conflating homonyms in the corresponding vector space. Bidirectional Encoder Representations from Transformers (BERT) [31] captures contextual relationships in a bidirectional way to contextualize the embedding of any given word based on the surrounding words. BERT is based on an encoder–decoder NN architecture, which can not only be used to generate word embeddings but can also be fine-tuned and further trained for various text mining tasks. For example, it has been used to model adverse event extraction as a named entity recognition (NER) task [11,32]. The topics of word embedding and BERT, in particular, will be revisited later in this paper in the context of motivating and describing our own approach to this problem.

The after-the-fact nature of text data collected from sources such as spontaneous reporting systems, medical literature, electronic health records, and social media naturally gives rise to postmarketing surveillance applications [2,33]. However, pharmacovigilance starts by collecting safety information derived from randomized controlled trials. Our review of text mining applications related to the identification of adverse events revealed that this source of data was underrepresented. This study addresses this gap by using SAE report forms collected during clinical trials as the primary source of data. Given that each trial focuses on a specific medicinal product, the problem is somewhat simplified as the need to extract information about the product itself is obviated. This also makes it more natural to define it as a multi-label text classification problem rather than an information extraction problem. Using the UMLS as our classification scheme, the main aim is to map each document to a set of coded adverse events. The main difficulty of the problem lies in differentiating between signs and symptoms associated with the underlying condition and those that represent adverse events. The fact that both types of references to signs and symptoms can be found within a single SAE report, often within the same sentence, renders a BOW approach unsuitable. Instead, we opt for a deep learning approach. Instead of LSTM approaches, which seem to dominate in our review of the related work, we opt for transformers, which tend to outperform recurrent NNs on a variety of natural language processing tasks.

## Methods

### Data Provenance

Data were provided by the Center for Trials Research (CTR), the largest group of academic (noncommercial) clinical trial staff in Wales. Their portfolio of work includes drug trials and complex interventions, mechanisms of disease and treatments, cohort studies, and informing policy and practice in partnerships with researchers across the United Kingdom and worldwide. Across all these trials, standard procedures are put in place to monitor and manage safety reporting and SAE in line with the regulatory requirements for research.

**Figure 1:**
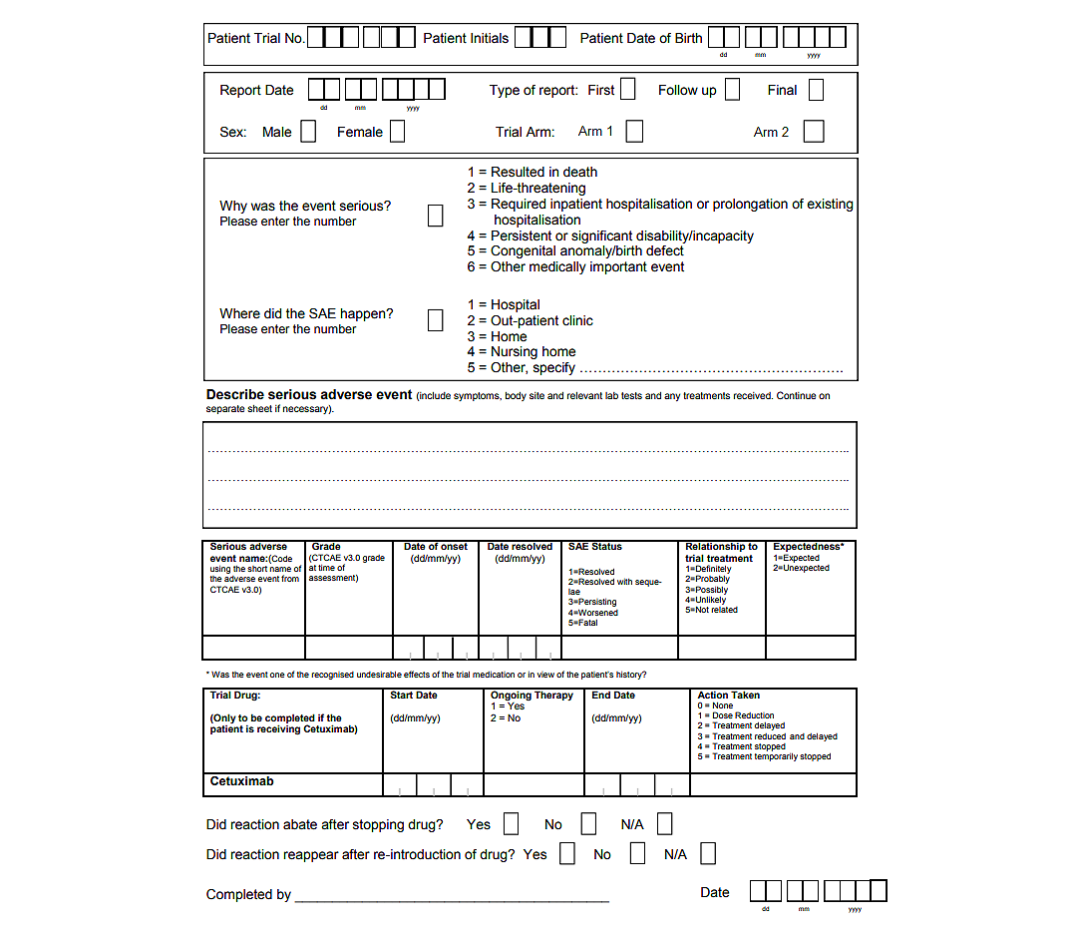
A serious adverse event (SAE) reporting form. CTCAE: Common Terminology Criteria for Adverse Events; N/A: Not Applicable.

Clinical trials SAE report forms (Figure 1) are completed by research nurses and physicians at hospital or clinical trial sites and submitted as PDF documents to the CTR central safety team for management and processing. They contain data on the SAE and a narrative description of the event. The narrative is used by the reviewer to help assess causal relationships with the trial drug but is not entered into the trial database and is not used in any analysis of the events. Completed SAE reports are then sent for review by a physician and, depending on the outcome of the review, are logged in the safety databases for the regulatory authorities, ethics committees, and drug companies.

Although narratives in noncommercial settings, such as CTR, can be digitized, this does not currently take place at the point of initial SAE reporting, as electronic data capture for the SAE report is associated with additional regulatory challenges, primarily because of the requirement for signature verification by a physician and a contemporaneous changelog. Clinical trial staff reviewing SAE reports are, thus, unable to systematically analyze the information provided in the narrative, missing an opportunity to identify the trends and potential safety signals. If the text mining approach were to identify additional safety events and signals not detected through standard reporting, processes could be altered to improve work practices at the level of a noncommercial CTU pharmacovigilance team.

This study aims to assess the feasibility of text mining in the context of such an analysis. The findings could affect the way regulatory narratives are reviewed and analyzed, for example, noncompliances or audit findings.

### Data Collection

Data were collected from 6 ongoing clinical trials, as described in Table 1.

Ethical review and approval were waived for this study as this study involved the use of secondary SAE data that were fully deidentified. All involved trials were conducted according to the guidelines of the Declaration of Helsinki and approved by the relevant research ethics committees. All chief investigators from these trials were consulted, and sponsor agreement was obtained for the use of the data in this secondary research study. Participant consent was also waived for the reasons stated above.

A subset of SAE reports was sampled randomly from each trial, giving a total of 286 reports. Phases 1 and 2 were early phases with a smaller number of participants and were not powered. The fewer numbers of reported SAEs were a function of the smaller numbers of participants compared with phase 3; hence, there were variations in the number of documents across the 6 trials.

The original SAE reports were pseudoanonymized at the point of extraction from the system by obscuring any links between the patient and their individual records. The narrative sections of the SAE reports were then transcribed and saved as Microsoft Word documents. The transcription process was extended to include deidentification by obscuring any personally identifiable information in a way that minimizes the risk of unintended disclosure of the identity of individuals and information about them. The transcribed documents were an average of 37 (SD 24) tokens long.

**Table 1:** Clinical trials from which data were collected.

ID	Description	Documents, n
Trial-1	A phase 2 study of neoadjuvant chemotherapy given before short-course preoperative radiotherapy as treatment for patients with MRI^a^-staged operable rectal cancer at high risk of metastatic relapse	5
Trial-2	A phase 1b/2 randomized placebo-controlled trial in postmenopausal women with advanced breast cancer previously treated with drug A	7
Trial-3	A randomized phase 3 clinical trial investigating the effect of drug B added to standard therapy in patients with lung cancer	131
Trial-4	Study of chemoradiotherapy in esophageal cancer, plus or minus drug C	34
Trial-5	A phase 1/2 single-arm trial to evaluate combination drugs for the treatment of advanced cancers, including first-line treatment of patients with advanced transitional cell carcinoma of the urothelium	3
Trial-6	A randomized phase 3, open-label, multicenter, parallel group clinical trial to evaluate and compare the efficacy, safety profile, and tolerability of oral drug X versus intravenous drug Y in the treatment of patients with breast cancer and bone metastases	106

^a^MRI: magnetic resonance imaging.

### Data Annotation

The aim of this task was to annotate adverse events in the transcribed versions of the SAE report forms. For the purpose of this task, an adverse event was defined as any unfavorable or unintended disease, sign, or symptom (including an abnormal laboratory finding) that is temporally associated with the use of a medical treatment or procedure, which may or may not be considered related to the medical treatment or procedure. Such an event could be related to the intervention, dose, route of administration, or patient or caused by an interaction with another drug or procedure.

The annotation guidelines prescribed the scope of the annotation task as follows: (1) focus only on adverse events that have occurred in the present or past, that is, ignore hypothetical or future events; (2) annotate the entire phrase that describes an adverse event; and (3) if the same adverse event were mentioned multiple times, then annotate every mention. The annotation process was based on the following instructions: (1) identify an adverse event that is mentioned in the narrative, (2) select the text that describes the adverse event, and (3) highlight the selected text.

The text editing operations were performed using Microsoft Word, which was preferred over a specifically designed annotation tool such as BRAT or Bionotate [34] because of zero installation and training overhead. Microsoft Word supports the bulk selection of text based on its formatting. This functionality was used to export highlighted text as standoff annotations, which were later used to calculate the interannotator agreement.

**Figure 2:**
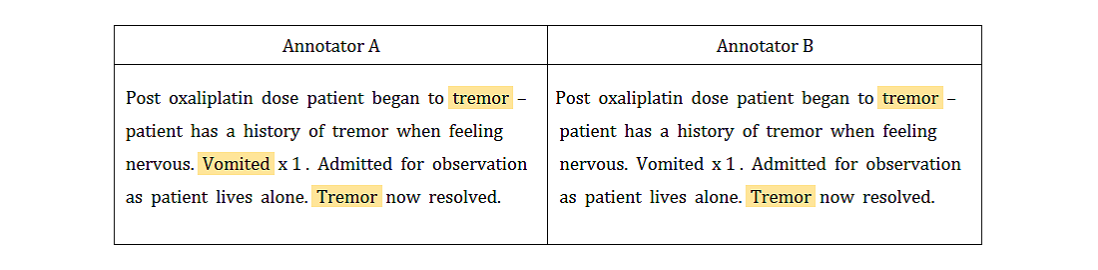
A serious adverse event report annotated independently by 2 annotators. The annotations are highlighted in yellow.

A total of 2 annotators independently annotated all the documents. Figure 2 provides an example. Here, both annotators annotated 2 mentions of tremor but did not annotate the historical mention of tremor as it was not temporally associated with the use of the medical treatment that was the subject of the given clinical trial. Further, 1 reviewer failed to annotate vomiting, leading to disagreement, which was later resolved through discussion. To identify all such cases, we compared all annotations automatically and measured the interannotator agreement.

The 2 annotators labeled SAEs as phrases, which were sequences of words whose total number, together with their start and end positions, were not prefixed. Comparing the interannotator agreement at the token level, as suggested by Tomanek et al [35], was not entirely appropriate for 2 reasons. First, the annotators labeled phrases as sequences of tokens instead of labeling the tokens individually. Therefore, such an approach approximated the original annotation task. More importantly, the number of negative cases (ie, the tokens that had not been annotated) would inevitably be much larger than the number of positive cases, thus skewing the data. The lack of a well-defined number of negative cases prevented the use of traditional interannotator agreement measures such as Cohen κ statistic [36]. A common way of quantifying interannotator agreement in such circumstances is to use information retrieval performance measures instead [37]. By treating one annotator’s annotations as the gold standard and the other one’s as predictions, we calculated the numbers of true positives (TPs), false positives (FPs), and true negatives, as shown in the confusion matrix (Table 2). When these values were combined to calculate the F1 score, it no longer mattered which annotator was considered the gold standard as this measure was symmetrical.

**Table 2:** Agreement between 2 annotators.

Positive or negative	Gold positive	Gold negative
Predicted positive	TP^a^=744	FP^b^=50
Predicted negative	FN^c^=98	N/A^d^

^a^TP: true positive.

^b^FP: false positive.

^c^FN: false negative.

^d^N/A: not applicable.

These values can then be used to calculate the precision (P), R, and F1-score as follows (where FN denotes false negative):

P=TP/(TP+FP)=744/(744+50)=0.9370

R=TP/(TP+FN)=744/(744+98)=0.8836

F1=(2×P×R)/(P+R)=0.9095

**Figure 3:**
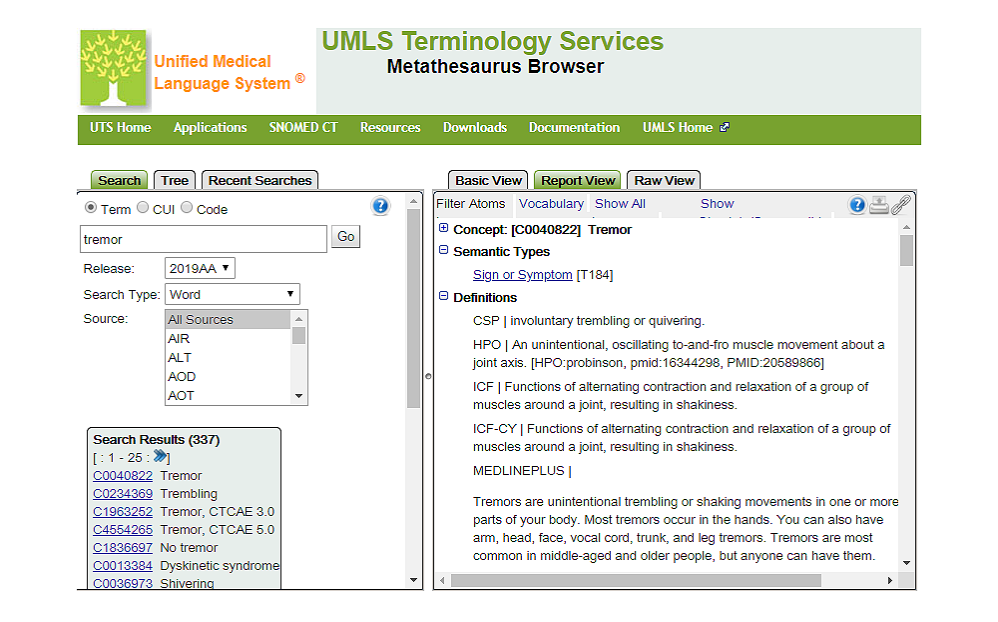
Metathesaurus browser search results.

**Figure 4:**
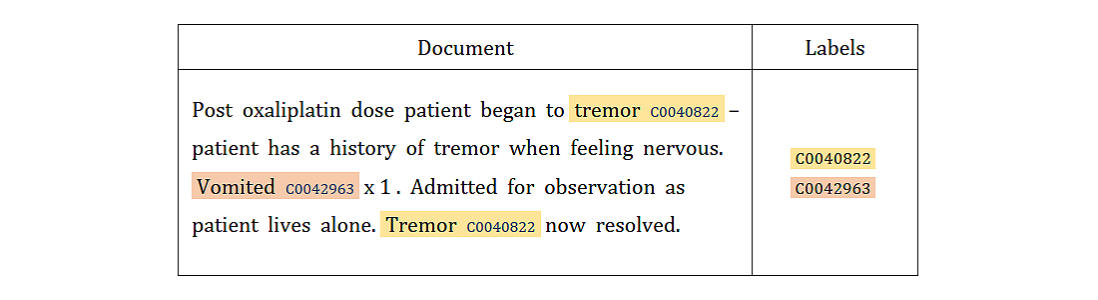
Coding of documents against the Uniﬁed Medical Language System.

An advantage of using information retrieval performance measures to estimate interannotator agreement is that their values can later be used to gauge a system against human-like performance. At F1=0.9095, the interannotator agreement was found to be relatively high. A total of 148 disagreements were resolved through discussions to establish the ground truth. As part of the discussions, the agreed annotations of adverse events were coded manually against the UMLS, which integrates multiple terminologies, classifications, and coding standards in an attempt to support the interoperability between biomedical information systems, including electronic health records [38]. The MetaThesaurus Browser, a web-based search interface, was used to query the UMLS for each annotation to identify the corresponding concept (Figure 3). This searching procedure involved checking concept definitions to make sure that the chosen concept matched the sense of the adverse event annotation. Each concept in the UMLS is assigned a concept unique identifier (CUI), which was used to code the corresponding annotation (see Figure 4 for examples). Subsequently, the CUI codes were extracted, duplicates were removed, and the remaining CUIs were used as class labels for each document. Table 3 provides a statistical summary of the annotated data set, which contains a total of 995 class labels.

**Table 3:** Statistical properties of the annotated data set.

Statistical properties	Document length (in tokens)	Annotations	Class labels
Values, minimum	2	1	1
Values, maximum	223	20	19
Values, median	31	3	3
Values, mean (SD)	36.71 (23.77)	3.76 (2.46)	3.48 (2.18)

### Problem Representation

The aim of this study was to automate the identification of adverse events described in the narrative section of the SAE reports. This goal was cast as a text classification problem. Given a document and classification scheme, the system should label the document with the relevant classes from the given scheme. In our case, the document was an SAE report, a classification scheme was the set of concepts encompassed by the UMLS, and their CUIs were used as class labels. The second column in Figure 4 provides an example of the expected output.

**Figure 5:**
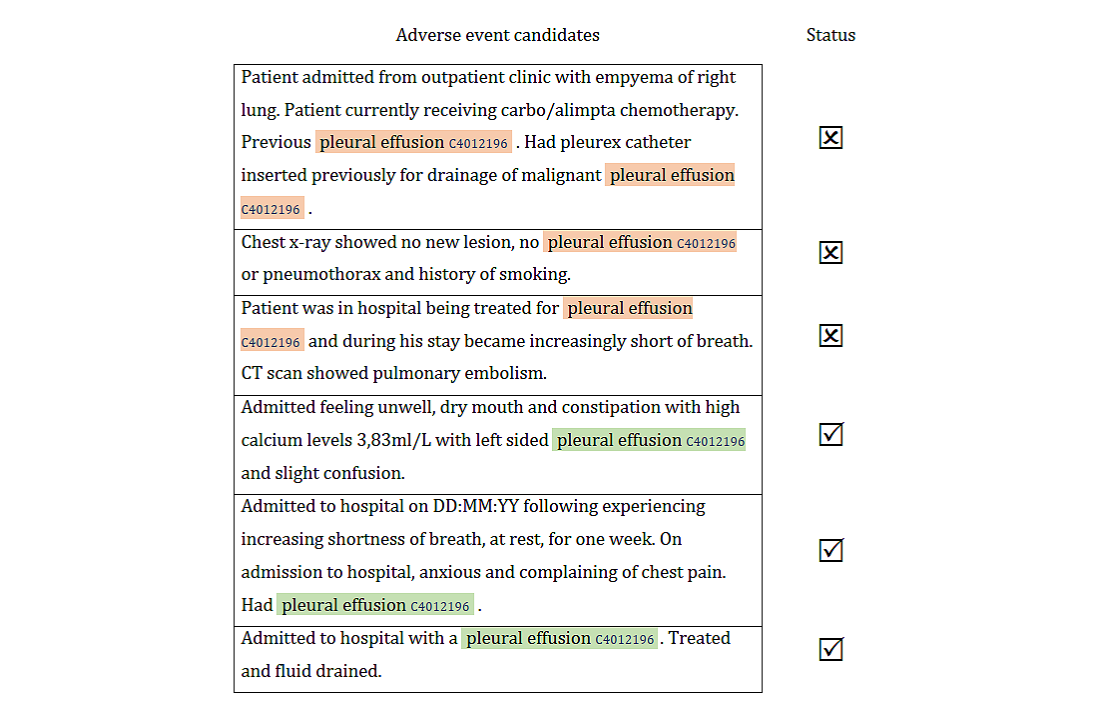
Adverse event identification as a binary classification task. CT: computed tomography.

To identify the possible adverse events mentioned in a document, the first step involved looking for concepts of the relevant semantic types. In our approach, the UMLS dictionary lookup was restricted to 6 manually selected semantic types: disease or syndrome, finding, injury or poisoning, neoplastic process, pathological function, and sign or symptom. Some of their mentions could be in the context of medical history and, therefore, not necessarily constitute an adverse event. To differentiate between the 2 types of mentions, we formulated a binary classification task at the concept level: given a context, does a specific UMLS concept constitute an adverse event? Figure 5 provides different references to the concept of *pleural effusion*. For example, the first 3 references do not constitute adverse events. The first and third mentions of *pleural effusion* refer to medical history, whereas the second mention is negated. The remaining 3 mentions of *pleural effusion* refer to the cause of hospital admissions that prompted SAE reporting.

**Figure 6:**
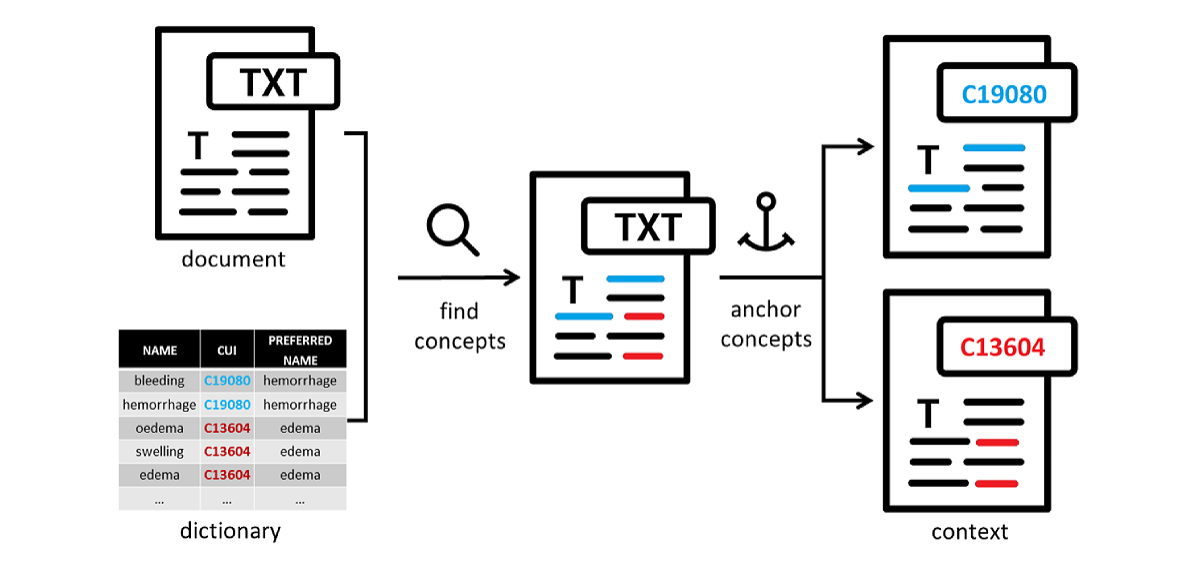
Identification of potential adverse event mentions. CUI: concept unique identifier.

The practical implementation of such problem representations started with linguistic preprocessing, which was originally developed to support cohort selection from hospital discharge summaries, adapted for this study [39]. This module involved text segmentation and basic string operations such as lowercasing, fully expanding enclitics and special characters, replacing a selected subset of words and phrases with their representatives, and, in particular, replacing acronyms and abbreviations with their full forms. Finally, the preprocessed documents were analyzed using MetaMap [40], a highly configurable dictionary lookup software, to find mentions of UMLS concepts from the 6 semantic types listed above. Figure 6 illustrates a portion of the UMLS dictionary and how it was matched against the input text. As the figure illustrates, a single document might contain multiple adverse events. To support the classification of one adverse event candidate at a time, a separate copy of the given document was saved for each candidate. Each copy anchored a single concept, which may have had multiple occurrences, by marking them up in line. In addition, the text was further regularized by replacing all the concepts with their preferred names. Concept anchoring provided a simple, uniform representation of the potential adverse events, which enabled us to train a single binary classifier based on the context surrounding the anchors.

### Classification Rationale

**Figure 7:**
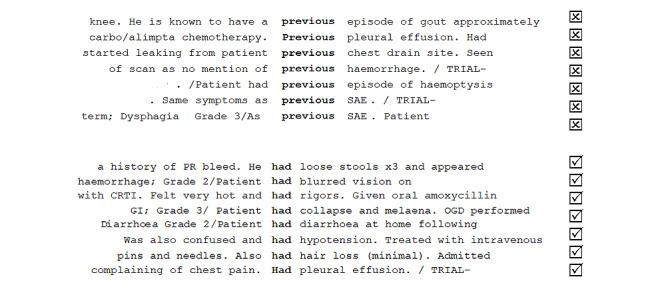
Observing the patterns of positive and negative modifiers. CRTI: common respiratory tract infection; GI: gastrointestinal; OGD: oesophagogastroduodenoscopy; PR: per rectum; SAE: serious adverse event.

**Figure 8:**
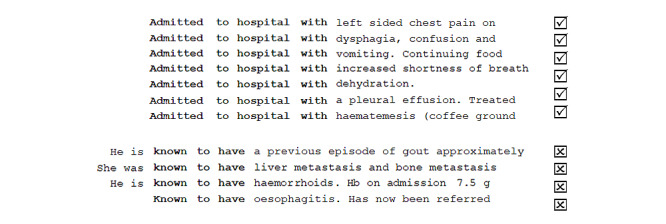
Observing more complex patterns of positive and negative use. Hb: hemoglobin.

The binary task formulation itself—*given a context, does a specific UMLS concept constitute an adverse event?*—indicates 2 main types of involved features: extrinsic (context) and intrinsic (concept). Extrinsic features may include the number of mentions within a document, the position within a document, and other words within a fixed-size window. When combined with gold standard annotations, machine learning can be used to discover how to differentiate between positive and negative contexts without having to manually describe the patterns of positive and negative use. For example, by considering the co-occurring words (see Figure 7 for examples) and the corresponding annotations, a simple NN can learn to use words such as *previous* and *have* as negative and positive modifiers, respectively. By considering a wider context, more complex patterns such as *admitted to hospital with* and *known to have* (see Figure 8 for examples) would start to emerge as positive and negative contexts, respectively. Traditionally, such patterns were observed using corpus linguistics methods, which were engineered manually and encoded formally as regular expressions [41]. In recent times, NNs are used to automatically capture both short- and long-range dependencies.

Similarly, lexical morphology could be explored in an NN approach to learn the patterns of subwords within a concept’s name, which were positively or negatively correlated with adverse events. For example, it is reasonable to expect that any concept identified as a potential adverse event that contains the word *chronic* (eg, *chronic obstructive airway disease* or *chronic infection*) is more likely to refer to a process than a single event. Similarly, any concept whose name contains a word *loss* (eg, *loss of appetite* or *hair loss*) is more likely to be an adverse event. The words themselves can be analyzed for affixes. For example, the prefix hypo- (low or below normal) can be used to increase the likelihood of concepts such as *hypocalcemia* or *orthostatic hypotension* corresponding to adverse events. Similarly, the suffix *-emia* (presence in the blood) can be used to identify concepts such as *cerebrovascular ischemia* or *hyperkalemia* as strong candidates for adverse events. Again, no prior medical knowledge is required to embed such features into NNs, which consider inputs and outputs simultaneously to support end-to-end learning and, hence, bypasses manual feature engineering.

### Text Representation

The first choice en route to implementing a binary adverse event classifier is text representation. Traditionally, the BOW representation, which is based on the frequency of occurrence of individual words, has been used to support text classification. Given that multiple signs and symptoms, some of which can be adverse events, are commonly discussed in an SAE report, the BOW representation would make it difficult to distinguish adverse events from other signs and symptoms discussed within the same document as it does not preserve local context. In addition, the BOW representation is not robust with respect to the out-of-dictionary problem; that is, any classifier trained using this representation will not be able to use words that were previously not encountered in the training data.

Word embedding can alleviate this problem. Word embedding is a mapping from the lexicosemantic space of words to the n-dimensional real-valued vector space. Methods such as word2vec [42] and GloVe [43] for learning word embeddings from large corpora rely on the hypothesis of distributional semantics, which claims that words occurring in similar contexts tend to convey similar meanings [44]. In other words, these methods assume that the meaning of a word depends on its context, that is, the frequency of co-occurrence with other words within a text window. Consequently, word embeddings tend to arrange semantically related words in similar spatial patterns. Therefore, by mapping a word to its embedding, it becomes possible to model its semantics numerically and thus use arithmetic operations to reason about it. This property is effectively used by NNs in which text is passed through a series of layers that each combines and transforms embeddings to eventually derive an output such as a class label in text classification or an answer in question answering.

Context-free word-embedding models such as word2vec [42] and GloVe [43] generate a single embedding for each word, making it impossible to differentiate between homonyms in the corresponding vector space. For example, the word *mole* would have a single embedding regardless of its many different meanings. Context-sensitive word-embedding models such as BERT [31] generate an embedding for each word based on the surrounding words. For example, the word *mole* used as *a unit of measurement* and *a disorder that affects the soft tissue* will have different representations in the word-embedding space.

BERT [31] is a transformer-based language model that captures contextual relationships in a bidirectional way. A transformer [45] is an encoder–decoder NN architecture that uses attention mechanisms to forward a holistic interpretation of a sequence to the decoder simultaneously rather than sequentially, as is the case in recurrent NNs such as LSTM and gated recurrent units. For each word, which is represented by its embedding, the self-attention layer considers other words, including their positions, in the same sentence to improve its encoding. As a workaround for the self-attention issue, BERT uses masked language modeling, that is, hides a certain percentage of the words using a special token [MASK] and uses their position to infer these words. The context-sensitive nature of BERT embeddings makes this language model perfectly suited for practical implementation of the classification rationale described earlier. In addition, BERT uses WordPiece tokenization to obtain subword units by applying a greedy segmentation algorithm to minimize the number of WordPieces in the training corpus [46]. This implies that the downstream classification model may be able to use the word morphology.

### Classification Model

The masked language modeling was 1 of the 2 tasks on which BERT was trained simultaneously. The second task was the next sentence prediction. In addition to [MASK], BERT uses 2 other special tokens for fine-tuning and specific task training: (1) a classification token [CLS], which indicates the beginning of a sequence and is commonly used for classification tasks (the output associated with this token is used for the next sentence prediction task); and (2) a sequence delimiter token [SEP], which indicates the end of a segment.

**Figure 9:**
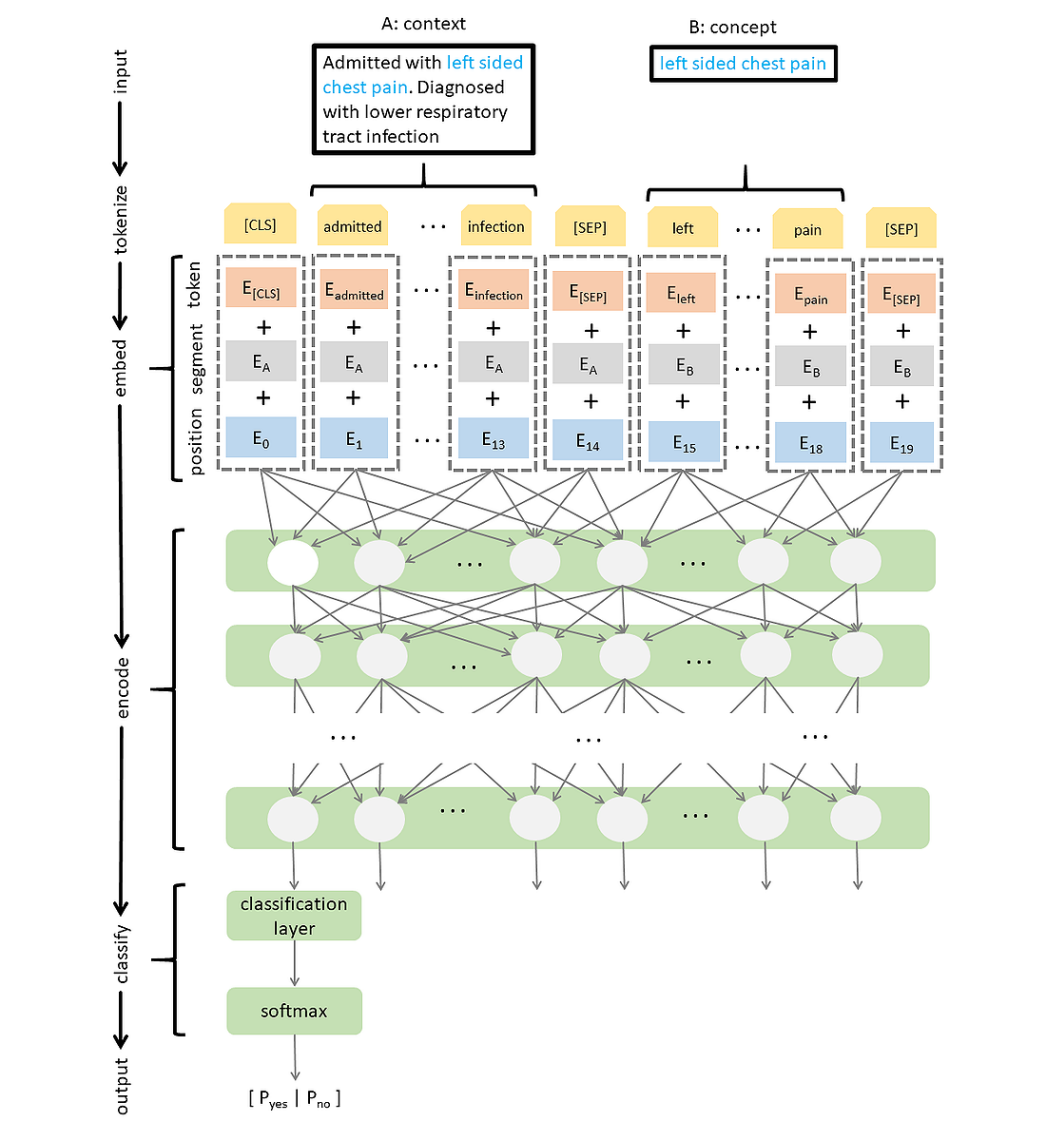
Architecture based on Bidirectional Encoder Representations from Transformer (BERT) for classification of adverse events. CLS: classification token; SEP: sequence delimiter token.

The embedding layer shown in Figure 9 illustrates the input format that BERT expects. Each token’s vocabulary identifier is mapped to a token embedding that is learned during training. Next, a binary vector is used to differentiate between 2 text segments, typically sentences. The type of segment depends on a specific task, for example, in question answering both question, and the reference text could be appended and separated by a special delimiter token [SEP]. In our model, we chose the anchored concept as one segment and its context (ie, the whole document) as another. The binary vector was mapped to a segment embedding using a lookup table, which was learned during training. Finally, local token positions were mapped to positional embeddings using a lookup table, which was updated during training.

The 3 types of embeddings were added and fed into the pretrained BERT_BASE_ model, which comprises 12 layers of transformer encoders, each having a hidden size of 768 and 12 attention heads. Each layer produces a token-specific output, which can be used as its (contextualized) embedding. Similar to binary classification tasks described in [31], the final transformer output corresponding to the special [CLS] token was taken as an aggregate problem representation, that is, pooled output, and passed on to the classification layer after a 0.1 dropout, which was used to reduce overfitting.

The classification layer reduced the size of the pooled output from 768 to 2, which corresponds to the log-odds (or logits) of the classification output with respect to the question of whether the given concept was an adverse event or not. In contrast to the network up to that point, the classification layer was not pretrained. Instead, the corresponding weights were learned during BERT fine-tuning. As suggested in the study by Devlin [31], the weights were initialized using a truncated normal distribution with mean 0 (SD 0.02). A softmax function was then applied to obtain the probability distribution of the 2 classes. The loss function (softmax cross entropy between the logits and the class labels) was optimized using the Adam optimizer with an initial learning rate of 2×10^–5^, which was chosen without any fine-tuning, based on the values suggested in the study by Devlin [31].

The classification model was trained for 8 epochs. This hyperparameter was preselected without any tuning. In each epoch, the training data were looped over in batches of 8 samples. The batch size was limited by memory. All other parameters were kept identical to those in the original BERT_BASE_ uncased model, including the clip norm of 1.0, and linear warmup (100 warmup steps with linear decay of learning rate). The system was implemented in TensorFlow [47], an open-source software library for machine learning, with a particular focus on training and inference of deep NNs, using the GeForce RTX 2080 (Nvidia Corp) graphics processing unit to accelerate deep learning.

## Results

During preprocessing, MetaMap was used to extract adverse event candidates. MetaMap failed to extract a total of 118 adverse events from the ground truth. Therefore, these instances automatically constituted FNs. The remaining 1021 adverse event candidates extracted by MetaMap were passed on to the BERT-based classification model shown in Figure 9. To understand the performance of the BERT classifier, we first focused only on these 995 adverse event candidates before amalgamating them with 118 FNs. Of the 995 candidates, 659 (66.2%) were positive instances (ie, regarded as adverse events in the ground truth), and 336 (33.8%) were negative instances (ie, not regarded as adverse events in the ground truth).

We performed 10 independent 5-fold cross-validations to evaluate the performance of the classification model. In other words, during each cross-validation, 20% of the documents were held out for evaluation, whereas the remaining 80% were used for training, and this was done 5 times in a row, each time using a different fold for evaluation. More specifically, for each of the 10 independent runs, we did the following:

The 286 unique document identifiers were first shuffled randomly and then split into 5 folds. Remember that each document may have contained multiple adverse event candidates, and a separate copy was created for each candidate during preprocessing. All copies of the same document shared the same document identifier; hence, there was no overlap of data across the folds. As the splitting was done by document irrespective of the number of events they contained, the actual number of samples (ie, potential adverse events identified by MetaMap) in each fold may vary. We looped over the folds, each time using a different fold for evaluation and the remaining 4 folds for training. Each time, we measured P, R, and F1 scores. Once each of the 5 folds was used for evaluation, we calculated the mean values obtained for each evaluation measure. Finally, these values were averaged over 10 independent runs.

The same cross-validation process was applied to the baseline approach. Remember that the goal of our system was to code adverse events against the UMLS; therefore, a UMLS lookup was inevitable. The lookup itself could be performed as the first step to identify an adverse event candidate (and code it at the same time) and then classify it. Alternatively, it could be performed as the last step to code an adverse event, which was first extracted from free text. In the former approach, we were dealing with a binary classification problem where it needed to be determined whether a given UMLS concept was an adverse event or not. In the latter approach, we were dealing with a sequence labeling problem where the boundaries of a token sequence that referred to an adverse event needed to be determined. This is how Du et al [32] approached the extraction of adverse events from safety reports by framing it as the NER problem and fine-tuning BERT for this task. We reimplemented and cross-validated their approach on our data set to establish the baseline. Although the authors originally used BERT for biomedical text mining (BioBERT) [48], we replaced it with BERT in our experiments to make their approach directly comparable with ours. The results achieved by the 2 contrasting approaches are presented in Table 4. Despite the similarities in the underlying technologies, we can observe a notable difference in the performance of the 2 approaches, most prominently in terms of P, where we can see an improvement of approximately 30 percent points over the baseline. A detailed analysis of this phenomenon is provided in the *Discussion* section. In this section, we proceed to describe the results achieved using our own approach.

**Figure 10:**
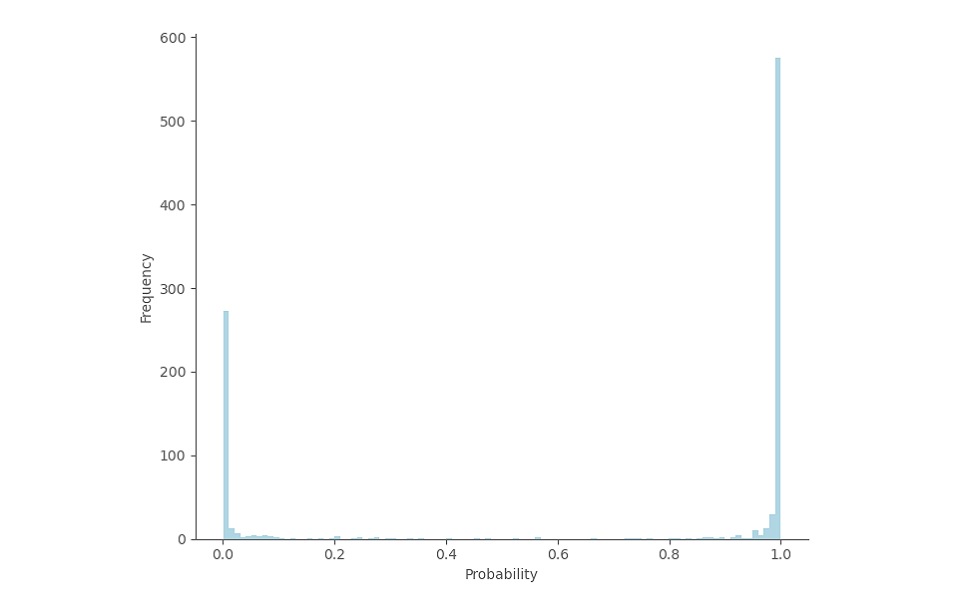
Distribution of prediction probabilities for all folds in a cross-validation experiment.

Figure 10 displays the distribution of the prediction probabilities. The histogram combines the predictions from all folds used for cross-validation. We can observe that most prediction probabilities are concentrated around the 2 extremes, 0 and 1, which suggests that the classification model is able to make clear-cut decisions, as it does not depend on a specific threshold.

**Table 4:** Evaluation results.

Parameters	Baseline approach: named entity recognition (BERT^a^)+concept extraction (MetaMap), mean (SD)	Our approach: concept extraction (MetaMap)+classification (BERT), mean (SD)
Precision	0.5715 (0.0076)	0.8638 (0.0057)
Recall	0.7116 (0.0096)	0.7604 (0.0121)
F1 score	0.6335 (0.0072)	0.8080 (0.0071)

^a^BERT: Bidirectional Encoder Representations from Transformers.

**Figure 11:**
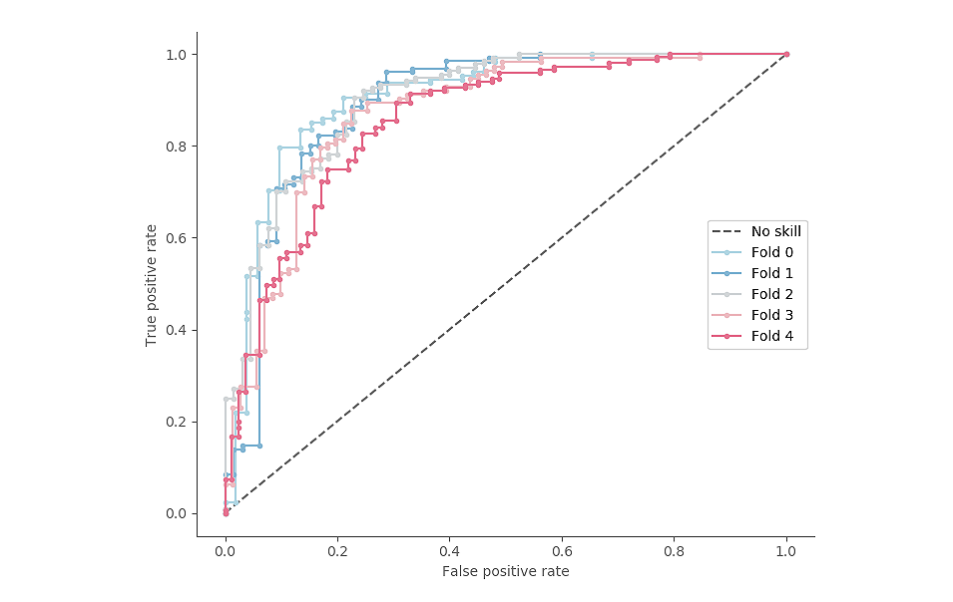
Receiver operating characteristic curve for each fold in a cross-validation experiment.

In Figure 11, we used receiver operating characteristic curves to illustrate the diagnostic ability of the classification model. A separate curve was provided for each of the 5 folds used for cross-validation. The plot shows the TP rate versus the FP rate at each classification threshold. The solid-colored lines correspond to the model’s performance, whereas the gray dashed line represents the performance of a classifier with no skill, that is, the one that always predicts the majority class. An ideal model would result in a curve that bows toward the coordinate (1,0). With its curve consistently lying close to the top-left corner, our model demonstrated very good classification performance. We summarized the receiver operating characteristic results by calculating the area under the curve to measure the ability of our model to distinguish between the 2 classes, with higher values indicating better performance. With an overall mean score of 0.8789 (SD 0.0101) and a range between 0 and 1, our model was clearly able to distinguish between adverse events and underlying conditions 87.79% of the time on average.

**Figure 12:**
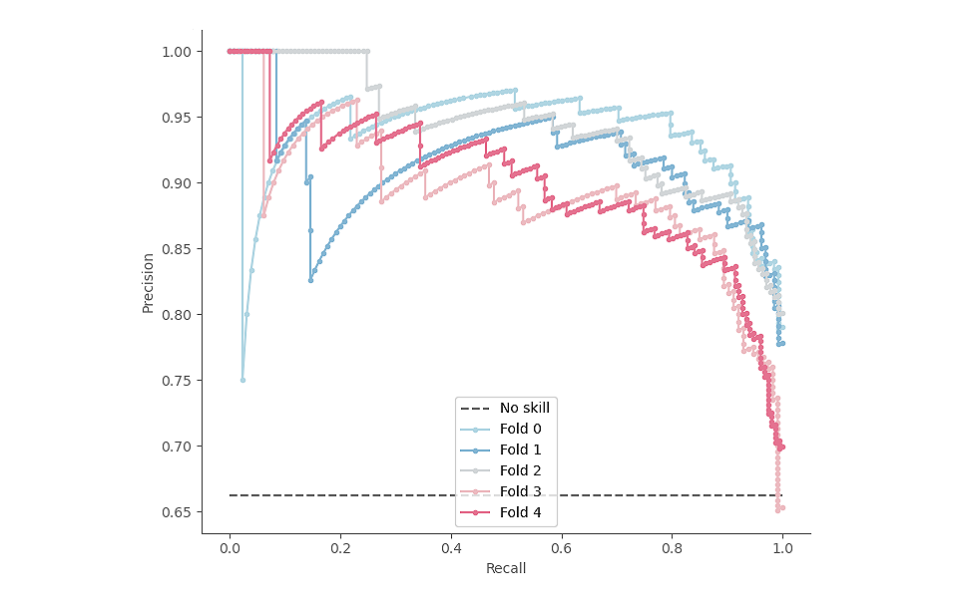
Precision-recall curve for each fold in a cross-validation experiment.

Finally, to account for the class imbalance, we also looked at the precision-recall (PR) curve shown in Figure 12. Again, the solid-colored lines correspond to our model’s performance, whereas the gray dashed horizontal line corresponds to a model with no skill, that is, a model whose P is equal to the proportion of positive samples. The PR curve of our model was relatively close to that of an ideal model, whose curve would bow toward the coordinate (1,1). In comparison to a no skill model, which would achieve a PR area under the curve score of 0.6533, our model reached a high score of 0.9108 (SD 0.0103), demonstrating its ability to correctly classify adverse events despite the class imbalance.

## Discussion

### Principal Findings

Previously, we provided details on calculating the interannotator agreement using P, R, and F1 score. When a system is evaluated against the ground truth, the corresponding values establish the human performance baseline, which in this case were P=0.9370, R=0.8836, and F1=0.9095. If we compare these values against the results provided in Table 4, we can observe a 10.15 percent points difference in the F1 score. In particular, we notice that the system’s R is 10.34 percent points lower than its P. There are 2 potential sources of type 2 errors in the system. Remember that the system first uses MetaMap to identify potential adverse events, which are then classified by BERT as positive or negative. Both components can give rise to FN results. First, any adverse event that MetaMap failed to forward to BERT would have been automatically counted as an FN. Second, any adverse event that MetaMap did supply to BERT for further classification could have still ended in an FN. MetaMap is a predefined rule-based system, and as such, its performance within our system is limited by external factors. BERT, on the other hand, has been trained for a specific task using the data set described here. Therefore, it is worth focusing specifically on its classification performance.

To evaluate how well BERT learned to classify adverse events, we removed those FNs from the ground truth that were never actually classified by BERT because of MetaMap failing to identify them in the first place. Table 5 provides the cross-validation results for BERT’s performance alone. We observe that the classification performance alone is much closer to the human performance baseline, lagging behind the F1 score by only 2.93 percent points.

**Table 5:** Bidirectional Encoder Representations from Transformers’ (BERT) performance.

Parameters	Named entity recognition (BERT), mean (SD)	Classification (BERT), mean (SD)
Precision	0.7484 (0.0066)	0.8651 (0.0053)
Recall	0.8237 (0.0086)	0.8974 (0.0104)
F1 score	0.7835 (0.0053)	0.8802 (0.0044)

If we now compare BERT’s classification performance given in Table 5 with the overall system performance given in Table 4, we can see that the P is virtually identical (0.8638 vs 0.8651), whereas R differs by 13.70 percent points (0.7604 vs 0.8974). Hence, we can conclude that the R of the overall system is primarily limited by MetaMap’s performance, which naturally raises the question of whether its use as a preprocessing step within our system was appropriate. The baseline method uses MetaMap as the postprocessing step; therefore, we investigated the extent of its effect on the overall performance by singling out BERT’s performance on the NER task, which was evaluated using the exact matching of phrases annotated in the ground truth. If we compare the first column of Table 5 with the second column of Table 4, we can observe that without MetaMap, BERT can certainly achieve higher R (0.8237 vs 0.7604) when it is allowed to determine the phrase boundaries on its own rather than having them prescribed by MetaMap.

Although such an approach is unarguably more flexible, it can also have a negative impact when the goal of the system is to code adverse events rather than only recognize their mentions in the text. If the phrase boundaries are not correctly detected as part of the NER task, then searching the UMLS using an incorrectly extracted phrase may provide an incorrect code. Consider, for example, 2 adverse events, *respiratory tract infection* (whose code in the UMLS is C0035243) and *urinary tract infection* (whose code is C0042029). Suppose that a system failed to correctly identify their boundaries, for example, by suggesting *tract infection* in both cases. The UMLS has no concept referring to *tract infection*; therefore, MetaMap would at best suggest *infection* (whose code is C3714514) as the closest concept matching the given search term, thus incorrectly coding both *respiratory tract infection* and *urinary tract infection*, resulting in 2 FNs (labeled C0035243 and C0042029 in the ground truth) and 2 FPs (both labeled C3714514 by the system). On the other hand, MetaMap can be configured to recognize the longest phrases from relevant semantic types and, in that way, impose tighter control of the process, reducing the number of both FPs and FNs. Although MetaMap may limit R, it does play an important role in controlling the P in our proposed approach, as the results in Table 4 clearly depict. Nonetheless, MetaMap could benefit from revising its rule-based dictionary lookup approach in light of the new advances in text mining and, in particular, deep learning approaches to bring its performance in line with the state of the art.

Focusing on BERT’s performance alone in Table 5, we can see that it performs better on the binary classification task than the NER task. This is not surprising, as the sequence labeling task is inherently more complex than binary classification. This is because of the number of possible sequences growing exponentially with the length of a document. In particular, the performance gap is bound to widen when training the corresponding models on a relatively small data set, as is the case in this study. Having <300 annotated documents available, we can see from Table 5 that BERT’s performance on the classification task is in the high 80s across all metrics, whereas its performance on the NER task is in the high 70s overall. This again justifies our choice to run BERT after MetaMap rather than the other way around.

Going back to the BERT’s classification performance provided in Table 5, while examining the misclassified examples, we noticed some patterns. Some simple negation patterns were not captured by the classifier. For example, in the document containing the sentence “Chest X-ray showed no new lesion, no pleural effusion disorder or pneumothorax and history of smoking,” both *pleural effusion disorder* and *pneumothorax* were misclassified as adverse events. Similarly, in the document with the sentence “admitted with right scaptula/back pain, no chest pain or dyspnea,” both *chest pain* and *dyspnea* were misclassified as adverse events.

This finding is in line with the current evidence that neural models struggle to generalize negation to out-of-sample data sets, even within the same domain [49]. The generalizability of negation remains a challenge, as none of the factors considered, including the annotation guidelines, the amount of data available, and their lexical and syntactic properties, fully explained the poor performance [50]. Empirical evidence suggests that the use of domain-specific embeddings such as BioBERT [48] may improve negation detection [51]. BERT can also be fine-tuned to support the negation detection task in clinical text [51,52]; however, this requires data to be annotated specifically for this task. Nonetheless, manual adaptation, be it rule modification or in-domain data annotation, remains a recommended strategy for optimizing performance in clinical natural language processing [50]. Rule-based systems for negation detection such as ConText [53] seem to transfer well within a domain [54]. Therefore, the simplest and most effective way of addressing negation as the source of errors in our proposed framework would be to use the ConText algorithm [53] to detect negated contexts and automatically exclude them from further consideration.

Some words, such as the word *decreasing*, can have the opposite effect depending on the context in which it is used. For example, *decreased mobility* implies a negative effect, whereas *decreased pain* implies a positive effect and not an adverse event. The system was not able to differentiate between such contexts. This could be remedied by incorporating domain knowledge about candidate adverse events. Alternatively, with a larger training data set, these properties could be learned directly from the data.

Finally, the classification model struggled when a given concept was used in multiple contexts. For example, for the concept *infection* in the document extract “admitted to hospital with lower respiratory tract infection [...] not commenced chemotherapy related infection,” the model misinterpreted the latter mention as a negated one and, consequently, misclassified this adverse event.

### Conclusions

This study established the feasibility of automated coding of adverse events described in the narrative section of the SAE reports. This, in turn, enables statistical analysis of adverse events and the patterns of such events so that any correlations with the use of medicines can be estimated in a timely fashion. An easy adaptation of an existing deep learning architecture trained on a relatively small data set demonstrates that similar tools can be built rapidly. In addition, the evaluation results show that such tools also perform with high accuracy. This performance can be attributed to the choice of the method. BERT is already pretrained on a large unlabeled corpus, which allows it to be fine-tuned on a small, labeled corpus for a specialized task. This is particularly relevant for clinical text mining applications, where the data annotation bottleneck has been identified as one of the key obstacles to machine learning approaches for clinical text mining [55].

Unfortunately, the relevant data are still mainly handwritten, which means that they cannot be immediately processed in the way proposed in this study. There are 2 ways in which this issue can be addressed. We can work with the stakeholders to change the policy on the means of collecting information on SAEs, for example, by transcribing the notes when they reach the safety and pharmacovigilance teams in the central trial unit, by requiring them to be typed, or by using some combination of these 2 approaches.

Alternatively, we can propose to develop methods to digitize handwritten notes automatically using tools such as Transkribus [56], which have been designed to digitize historical documents and allow the training of specific text recognition models. This would have a great potential for impact on safety by digitizing and mining legacy data from previous trials, where some medicinal products may have already reached the market, thus exposing the population to previously overlooked safety concerns. Currently, these issues prevent a systematic analysis of the information provided in the narrative of SAE reports, hence missing an opportunity to identify potential safety signals.

## Abbreviations

BERT: Bidirectional Encoder Representations from Transformers
BioBERT: Bidirectional Encoder Representations from Transformers for biomedical text mining
BOW: bag of words
CRF: conditional random field
CTR: Center for Trials Research
CTU: clinical trial unit
CUI: concept unique identifier
FN: false negative
FP: false positive
LSTM: long short-term memory
NER: named entity recognition
NN: neural network
PR: precision-recall
SAE: serious adverse event
TP: true positive
UMLS: Uniﬁed Medical Language System

## References

[1] (2018). Data Mining at FDA - White Paper. US Food and Drug Administration.

[2] Wong A, Plasek JM, Montecalvo SP, Zhou L (2018). Natural language processing and its implications for the future of medication safety: a narrative review of recent advances and challenges. Pharmacotherapy.

[3] Botsis T, Nguyen MD, Woo EJ, Markatou M, Ball R (2011). Text mining for the Vaccine Adverse Event Reporting System: medical text classification using informative feature selection. J Am Med Inform Assoc.

[4] Chee BW, Berlin R, Schatz B (2011). Predicting adverse drug events from personal health messages. AMIA Annu Symp Proc.

[5] Botsis T, Buttolph T, Nguyen MD, Winiecki S, Woo EJ, Ball R (2012). Vaccine adverse event text mining system for extracting features from vaccine safety reports. J Am Med Inform Assoc.

[6] Han L, Ball R, Pamer C, Altman R, Proestel S (2017). Development of an automated assessment tool for MedWatch reports in the FDA adverse event reporting system. J Am Med Inform Assoc.

[7] Iqbal E, Mallah R, Rhodes D, Wu H, Romero A, Chang N, Dzahini O, Pandey C, Broadbent M, Stewart R, Dobson RJ, Ibrahim ZM (2017). ADEPt, a semantically-enriched pipeline for extracting adverse drug events from free-text electronic health records. PLoS One.

[8] Roberts K, Demner-Fushman D, Tonning JM (2017). Overview of the TAC 2017 adverse reaction extraction from drug labels track. Proceedings of the Text Analysis Conference (TAC).

[9] Nikfarjam A, Sarker A, O'Connor K, Ginn R, Gonzalez G (2015). Pharmacovigilance from social media: mining adverse drug reaction mentions using sequence labeling with word embedding cluster features. J Am Med Inform Assoc.

[10] Cocos A, Fiks A, Masino A (2017). Deep learning for pharmacovigilance: recurrent neural network architectures for labeling adverse drug reactions in Twitter posts. J Am Med Inform Assoc.

[11] Fan Y, Zhou S, Li Y, Zhang R (2021). Deep learning approaches for extracting adverse events and indications of dietary supplements from clinical text. J Am Med Inform Assoc.

[12] Duke J, Friedlin J (2010). ADESSA: a real-time decision support service for delivery of semantically coded adverse drug event data. AMIA Annu Symp Proc.

[13] Combi C, Zorzi M, Pozzani G, Arzenton E, Moretti U (2019). Normalizing spontaneous reports into MedDRA: some experiments With MagiCoder. IEEE J Biomed Health Inform.

[14] Emadzadeh E, Sarker A, Nikfarjam A, Gonzalez G (2018). Hybrid semantic analysis for mapping adverse drug reaction mentions in tweets to medical terminology. AMIA Annu Symp Proc.

[15] Nikfarjam A, Gonzalez GH (2011). Pattern mining for extraction of mentions of Adverse Drug Reactions from user comments. AMIA Annu Symp Proc.

[16] Sarker A, Gonzalez G (2015). Portable automatic text classification for adverse drug reaction detection via multi-corpus training. J Biomed Inform.

[17] Liu J, Zhao S, Zhang X (2016). An ensemble method for extracting adverse drug events from social media. Artif Intell Med.

[18] Wang X, Hripcsak G, Markatou M, Friedman C (2009). Active computerized pharmacovigilance using natural language processing, statistics, and electronic health records: a feasibility study. J Am Med Inform Assoc.

[19] Skentzos S, Shubina M, Plutzky J, Turchin A (2011). Structured vs. unstructured: factors affecting adverse drug reaction documentation in an EMR repository. AMIA Annu Symp Proc.

[20] Hazlehurst B, Naleway A, Mullooly J (2009). Detecting possible vaccine adverse events in clinical notes of the electronic medical record. Vaccine.

[21] Negi K, Pavuri A, Patel L, Jain C (2019). A novel method for drug-adverse event extraction using machine learning. Informatics Med Unlocked.

[22] Wang C, Lin P, Cheng C, Tai S, Kao Yang Y, Chiang J (2019). Detecting potential adverse drug reactions using a deep neural network model. J Med Internet Res.

[23] Tao C, Lee K, Filannino M, Buchan K, Lee K, Arora T Extracting and normalizing adverse drug reactions from drug labels. Semantic Scholar.

[24] Cocos A, Masino A (2017). Combining rule-based and neural network systems for extracting adverse reactions from drug labels. Proceedings of the 2017 Text Analysis Conference, TAC 2017.

[25] Belousov M, Milosevic N, Dixon W, Nenadic G (2019). Extracting adverse drug reactions and their context using sequence labelling ensembles in TAC2017. Proceedings of the 2017 Text Analysis Conference, TAC 2017.

[26] Dandala B, Mahajan D, Devarakonda M (2017). IBM Research system at TAC 2017: adverse drug reactions extraction from drug labels. Proceedings of the 2017 Text Analysis Conference, TAC 2017.

[27] Sun J, Gu X, Ding C, Li C, Li Y, Li S, Xu W (2017). BUPT-PRIS system for TAC 2017 event nugget detection, event argument linking and ADR tracks. Proceedings of the 2017 Text Analysis Conference, TAC 2017,.

[28] Tiftikci M, Özgür A, He Y, Hur J (2017). BUPT-PRIS System for TAC 2017 Event Nugget Detection, Event Argument Linking and ADR Tracks. Proceedings of the 2017 Text Analysis Conference, TAC 2017.

[29] Xu J, Lee H-J, Ji Z, Wang J, Wei Q, Xu H (2017). UTH CCB system for adverse drug reaction extraction from drug labels at TAC-ADR 2017. Proceedings of the 2017 Text Analysis Conference, TAC 2017.

[30] Pawar S, Palshikar G, Bhattacharyya P, Ramrakhiyani N, Gupta S, Varma V (2017). TCS Research at TAC 2017: Joint extraction of entities and relations from drug labels using an ensemble of neural networks. Proceedings of the 2017 Text Analysis Conference, TAC 2017.

[31] Devlin J, Lee M, Toutanova K (2018). BERT: pre-training of deep bidirectional transformers for language understanding. arXiv.

[32] Du J, Xiang Y, Sankaranarayanapillai M, Zhang M, Wang J, Si Y, Pham HA, Xu H, Chen Y, Tao C (2021). Extracting postmarketing adverse events from safety reports in the vaccine adverse event reporting system (VAERS) using deep learning. J Am Med Inform Assoc.

[33] Luo Y, Thompson WK, Herr TM, Zeng Z, Berendsen MA, Jonnalagadda SR, Carson MB, Starren J (2017). Natural language processing for EHR-based pharmacovigilance: a structured review. Drug Saf.

[34] Neves M, Leser U (2014). A survey on annotation tools for the biomedical literature. Brief Bioinform.

[35] Tomanek K, Hahn U (2021). Proceedings of the Linguistic Annotation Workshop; Suntec, Singapore2009.

[36] Deleger L, Li Q, Lingren T, Kaiser M, Molnar K, Stoutenborough L, Kouril M, Marsolo K, Solti I (2012). Building gold standard corpora for medical natural language processing tasks. AMIA Annu Symp Proc.

[37] Hripcsak G, Rothschild AS (2005). Agreement, the f-measure, and reliability in information retrieval. J Am Med Inform Assoc.

[38] Bodenreider O (2004). The Unified Medical Language System (UMLS): integrating biomedical terminology. Nucleic Acids Res.

[39] Spasic I, Krzeminski D, Corcoran P, Balinsky A (2019). Cohort selection for clinical trials from longitudinal patient records: text mining approach. JMIR Med Inform.

[40] Aronson AR, Lang F (2010). An overview of MetaMap: historical perspective and recent advances. J Am Med Inform Assoc.

[41] Spasic I, Sarafraz F, Keane JA, Nenadic G (2010). Medication information extraction with linguistic pattern matching and semantic rules. J Am Med Inform Assoc.

[42] Mikolov T, Sutskever I, Chen K, Corrado G, Dean J (2013). Distributed representations of words and phrases and their compositionality. Adv Neur Inf Process Syst.

[43] Pennington J, Socher R, Manning C (2014). GloVe: global vectors for word representation. Proceedings of the Conference on Empirical Methods in Natural Language Processing (EMNLP).

[44] Harris ZS (2015). Distributional structure. Word.

[45] Vaswani A, Shazeer N, Parmar N, Uszkoreit J, Jones L, Gomez A (2017). Attention is all you need. ArXiv.org.

[46] Wu Y, Schuster M, Chen Z, Le Q, Norouzi M, Macherey W (2016). Google's neural machine translation system: bridging the gap between human and machine translation. ArXiv.org.

[47] Abadi M, Barham P, Chen J, Chen Z, Davis A, Dean J (2016). Tensorflow: a system for large-scale machine learning. Proceedings of the 12th USENIX conference on Operating Systems Design and Implementation.

[48] Lee J, Yoon W, Kim S, Kim D, Kim S, So C, Kang J (2020). BioBERT: a pre-trained biomedical language representation model for biomedical text mining. Bioinformatics.

[49] Grivas A, Alex B, Grover C, Tobin R, Whiteley W (2020). Not a cute stroke: analysis of rule- and neural network-based information extraction systems for brain radiology reports. Proceedings of the 11th International Workshop on Health Text Mining and Information Analysis at the Conference on Empirical Methods in Natural Language Processing.

[50] Wu S, Miller T, Masanz J, Coarr M, Halgrim S, Carrell D, Clark C (2014). Negation's not solved: generalizability versus optimizability in clinical natural language processing. PLoS One.

[51] Rivera Zavala R, Martinez P (2020). The impact of pretrained language models on negation and speculation detection in cross-lingual medical text: comparative study. JMIR Med Inform.

[52] Lin C, Bethard S, Dligach D, Sadeque F, Savova G, Miller T (2020). Does BERT need domain adaptation for clinical negation detection?. J Am Med Inform Assoc.

[53] Harkema H, Dowling JN, Thornblade T, Chapman WW (2009). ConText: an algorithm for determining negation, experiencer, and temporal status from clinical reports. J Biomed Inform.

[54] Sykes D, Grivas A, Grover C, Tobin R, Sudlow C, Whiteley W, Mcintosh A, Whalley H, Alex B (2020). Comparison of rule-based and neural network models for negation detection in radiology reports. Nat Lang Eng.

[55] Spasic I, Nenadic G (2020). Clinical text data in machine learning: systematic review. JMIR Med Inform.

[56] Kahle P, Colutto S, Hackl G, Mühlberger G (2017). Transkribus - A service platform for transcription, recognition and retrieval of historical documents. Proceedings of the 14th IAPR International Conference on Document Analysis and Recognition (ICDAR); Kyoto, Japan.

